# Infective Endocarditis Masked by Narrow Focus Thinking, Inadequate Physical Examination and Analgesic Medication

**DOI:** 10.7759/cureus.5645

**Published:** 2019-09-13

**Authors:** Larry Nichols, Michel Hernandez, John H Henderson IV

**Affiliations:** 1 Pathology, Mercer University School of Medicine, Macon, USA; 2 Biology, Mercer University School of Medicine, Macon, USA; 3 Internal Medicine, Mercer University School of Medicine, Macon, USA

**Keywords:** infective endocarditis, osler nodes, medical error, physical examination, medical error of omission, janeway lesions, diagnostic error, medical history taking, patient safety

## Abstract

It is important not to miss a diagnosis of infective endocarditis. It is fatal if untreated and most often successfully treatable if recognized. We report a classic case of subacute bacterial endocarditis with Osler nodes and Janeway lesions due to viridans streptococci from an oral source of poor dentition, infecting a presumably abnormal mitral valve. The diagnosis was missed repeatedly by multiple different clinicians over the course of seven months. Detailed analysis of this case suggests some of the pitfalls that led to the delay in diagnosis. The infective endocarditis was masked by analgesic medication, inadequate physical examination, and narrow focus thinking. The images of this case can serve as a reminder of the features of infective endocarditis. The detailed history and discussion can provide potential lessons in how to not miss the diagnosis of infective endocarditis.

## Introduction

It is essential not to miss a diagnosis of infective endocarditis. Undiagnosed and untreated, it is uniformly fatal. Diagnosed and treated, approximately two-thirds of patients with infective endocarditis can be saved. The key to improving the outcomes of infective endocarditis is early diagnosis [1]. The key to early diagnosis of infective endocarditis is history and physical examination, as it is for most diseases [2]. In the history and physical examination, the hallmarks of infective endocarditis are fever and heart murmur [3]. Analgesic medication with non-steroidal anti-inflammatory drugs or acetaminophen can mask fever. Heart murmur can be absent, especially in children, who have a murmur reported in only 10-20% of cases [4]. Heart murmur can be missed due to inadequate physical examination [5]. Osler nodes, Janeway lesions, and Roth spots are more specific for endocarditis but are rare manifestations of it [3]. When Osler nodes and Janeway lesions are present, their link to an underlying diagnosis of endocarditis can be missed, especially if a clinician’s thinking is narrowly focused on some other manifestation of infective endocarditis. This case is reported to convey these important clinical lessons.

## Case presentation

A 22-year-old female college student began experiencing tender fingertips, one or two at a time, in August. She had an episode in early October of sudden onset numbness followed by pain in her proximal right forearm, associated with tenderness on deep palpation. Throughout October, she had episodes of soreness in rotating sites including the back of her head, her abdomen, her right thigh and right calf. Pain in her dorsal left foot awoke her in the middle of the night in late October. She woke up with a painful swollen right wrist (“felt like it was broken”), making it difficult to write, in early November.

The patient presented on November sixth to a nurse practitioner in a public health clinic with a chief complaint of intermittent muscle pain. She denied fever, chills, fatigue, bleeding or skin rash. Her temperature was 36.9 °C (98.5 °F), pulse 104 beats/minute, blood pressure 112/76 mmHg, and respirations 18 breaths/minute. She had normal heart sounds with no murmur heard, and normal peripheral pulses, musculoskeletal and neurological examination. Her white blood cell count was 7,800/cu mm (79% neutrophils) and hemoglobin 12.6 g/dL; platelets, blood urea nitrogen, creatinine, sodium, potassium, chloride, bicarbonate, calcium, glucose, bilirubin, alkaline phosphatase, alanine aminotransferase (ALT), aspartate aminotransferase (AST), albumin and total protein were all normal. She was diagnosed with “sensory disturbance (primary)” and given no treatment.

**Figure 1:**
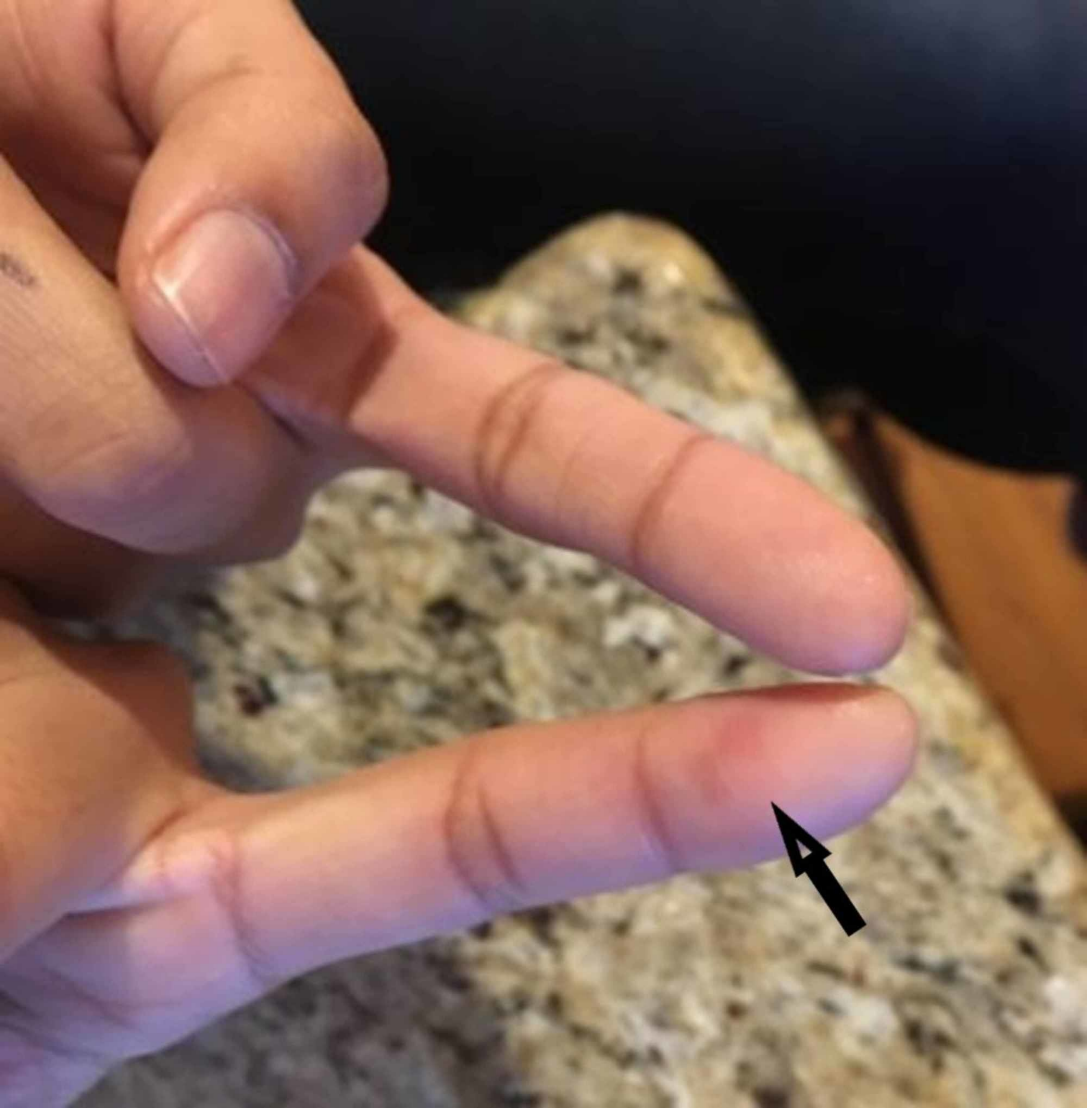
Osler nodes, sites of transient tenderness in finger pads, one with “skin blotching” (arrow)

Over the next two months, the patient continued to have episodes of pain and tenderness for which she took over the counter ibuprofen and acetaminophen. She had an episode of “skin blotching” on the pad of a finger that she photographed⁠-one of the fingertips where she had been having transient tenderness before taking over the counter analgesic medications (Figure 1). She denied fever, chills or fatigue. She was seen again on January third and was noted to be tearful and “worried about everything”. Her temperature was 36.5 °C (97.7 °F), pulse 112 beats/minute, blood pressure 128/80 mmHg, and respirations 18 breaths/minute. She had no murmur heard, and unchanged physical examination. She was diagnosed with a generalized anxiety disorder for which duloxetine was prescribed, and myalgia for which over the counter ibuprofen and acetaminophen were recommended.

The patient continued to have episodes of pain and tenderness for which she took ibuprofen and acetaminophen, but she discontinued taking duloxetine. She was seen in a hospital emergency department on January 18 with a chief complaint of “hurting all over with pain in fingers, toes, inflammation, swelling” and fingers going numb for six months. Her temperature was 36.6 °C (97.8 °F), pulse 111 beats/minute, and blood pressure 119/73 mmHg. Her white blood cell count was 9,580/cu mm (76% neutrophils), hemoglobin 10.9 g/dL and C-reactive protein 9.36 mg/dL (reference range, 0.02-0.50). She was discharged from the emergency department with a diagnosis of “multiple musculoskeletal pains-swelling” for which methocarbamol was prescribed.

**Figure 2:**
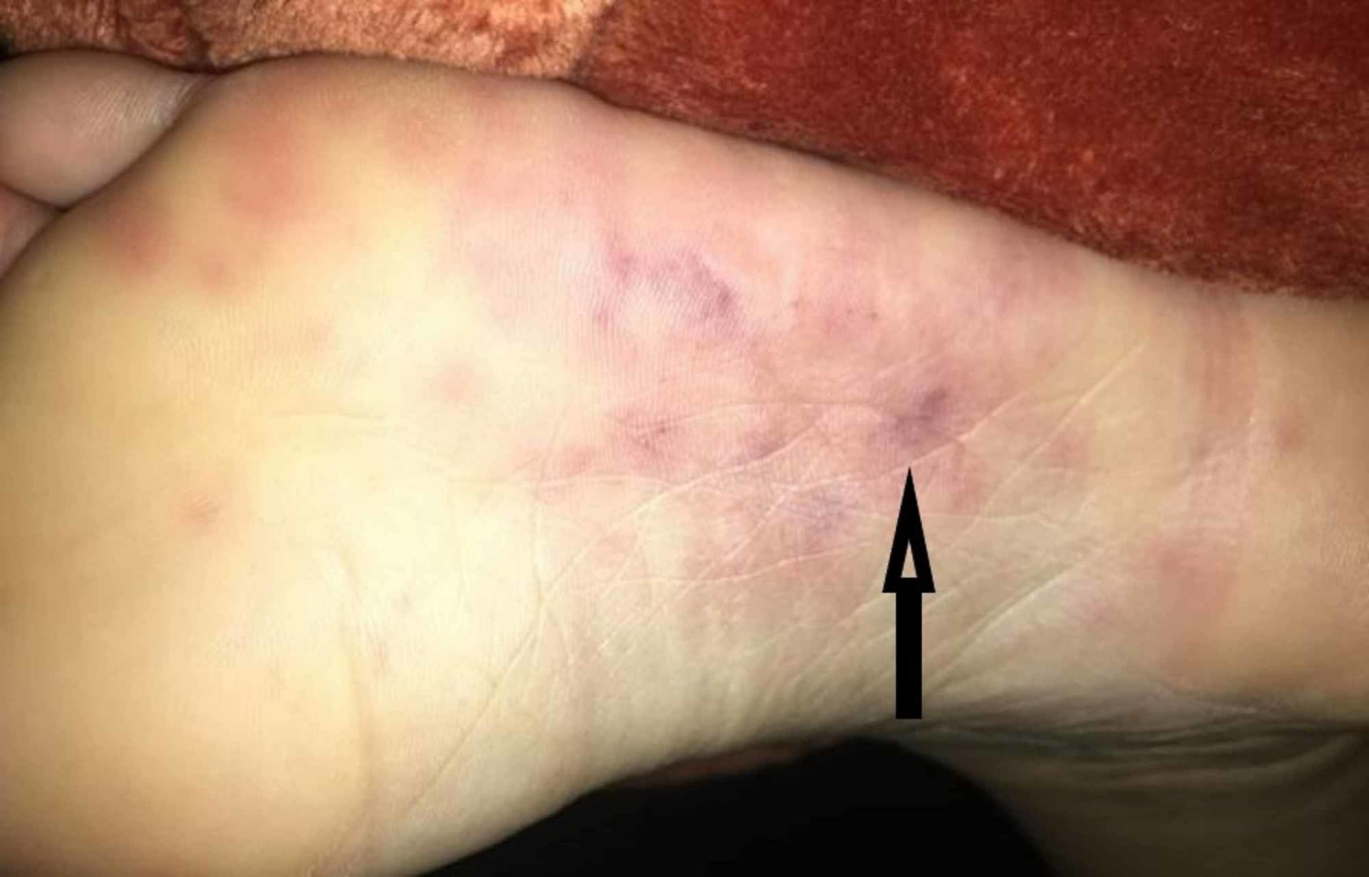
Janeway lesions, erythematous macules, some coalescing, associated with slightly nodular hemorrhages (arrow)

The patient had an episode of paresthesia (tingling) on her upper back, an episode of left facial numbness and an episode of vertigo. She had an episode of skin blotching on her soles, which were tender, on January 30, that she photographed (Figure 2).

The patient was seen in the emergency department on February 13 with sudden onset left flank pain, temperature 36.5 °C (97.7 °F), pulse 120 beats/minute, blood pressure 114/97 mm Hg and respirations 18 breaths/minute. She had normal heart sounds with no murmur heard, but tenderness over the left kidney. Her white blood cell count was 15,250/cu mm (85% neutrophils), hemoglobin 9.9 g/dL and albumin 3.2 g/dL; platelets, blood urea nitrogen, creatinine, sodium, potassium, chloride, bicarbonate, calcium, glucose, bilirubin, alkaline phosphatase, ALT, AST, lipase and total protein were all normal. Urinalysis showed 15 white blood cells/high power field, 27 red blood cells/high power field and occasional bacteria. Abdominal computed tomography showed no abnormalities. Pyelonephritis was diagnosed and treated with morphine, acetaminophen-oxycodone, and ceftriaxone. Urine culture was ordered, but not obtained before the patient was discharged with prescriptions for tramadol, ibuprofen, and ciprofloxacin.

The patient presented on February 20 to the nurse practitioner in the public health clinic with episodes of moderate dizziness, a sensation of the room spinning with moving her head, lasting for minutes, associated with nausea, intermittently over the previous two weeks. The worst episode occurred that day and prompted her visit to the clinic. The patient denied headache, fever, chills, ear pain, teeth pain, sore throat or anxiety. Her temperature was 36.8 °C (98.2 °F), pulse 86 beats/minute, blood pressure 118/84 mm Hg and respirations 18 breaths/minute. While listening to the patient’s heart, the nurse asked her if she had ever had a heart murmur. The patient was unaware that she had and replied no. The nurse recorded hearing no murmur. Vertigo was diagnosed and the patient was prescribed meclizine.

The patient was admitted to a community hospital in another city on March fourth with sudden onset epigastric pain, associated with nausea, vomiting, and weight loss (3.6 kg). She had been lightheaded for several days and had had hematochezia for three days, three weeks prior. Her temperature was 36.6 °C (97.9 °F), pulse 104 beats/minute, blood pressure 116/79 mmHg, respirations 18 breaths/minute and oxygen saturation 96% on room air. She had epigastric tenderness and stool positive for occult blood. “No murmur heard” was reported by the admitting physician and no murmur was reported by any of the multiple other physicians on the case. The patient’s hemoglobin was 8.7 g/dL, mean corpuscular volume 83 fL, platelets 405,000/cu mm, and white blood cell count 11,600/cu mm (82% neutrophils). She had been taking ibuprofen, 1600 mg/day. Erosive gastritis was diagnosed by endoscopy and her anemia was attributed to acute blood loss from ibuprofen. She was discharged March fifth on pantoprazole, sucralfate, iron, and tramadol. Gastric biopsy was subsequently seen to show no abnormalities.

**Figure 3:**
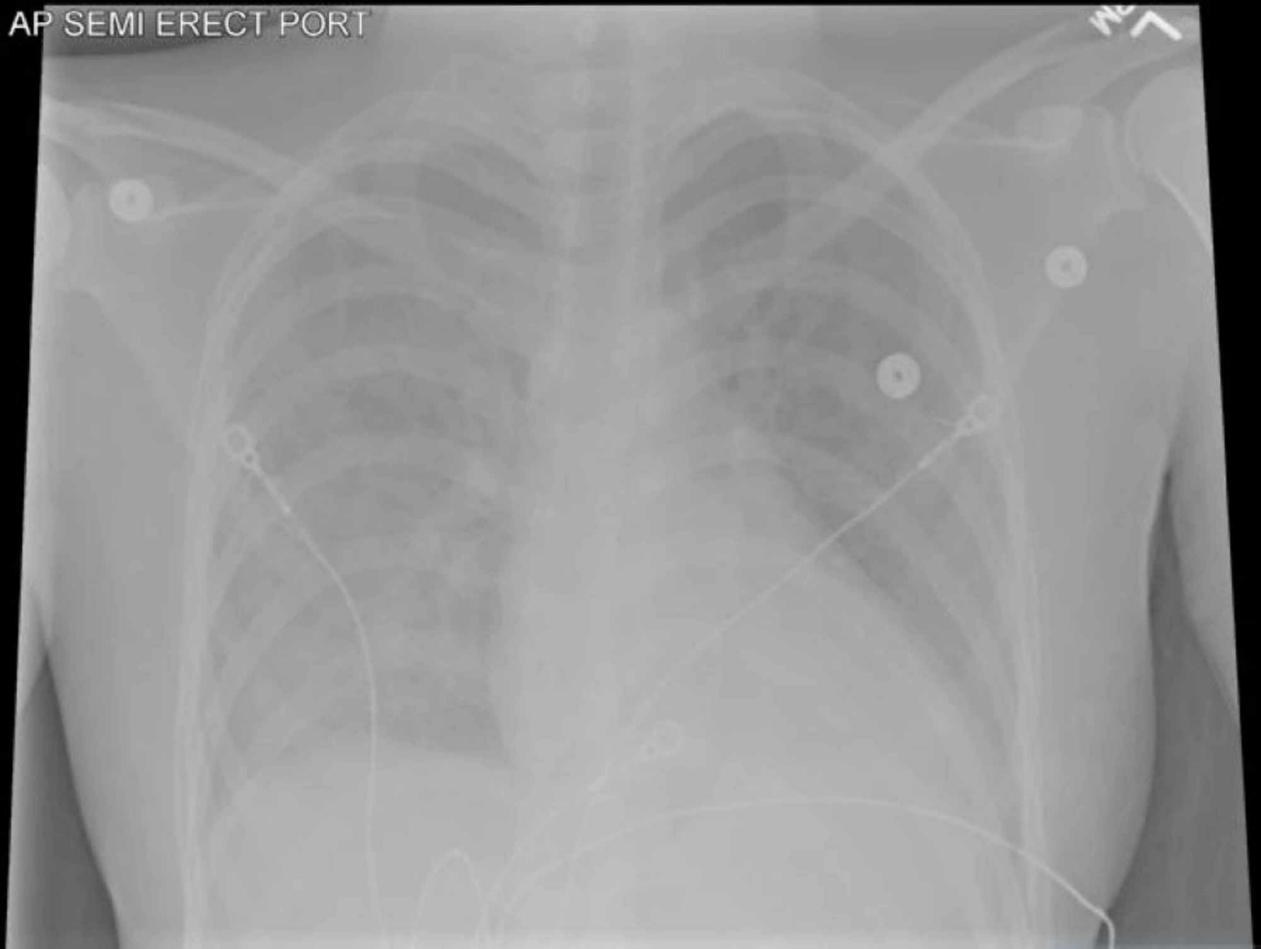
Portable antero-posterior chest radiograph showing diffuse ground-glass opacity in the right lung

**Figure 4:**
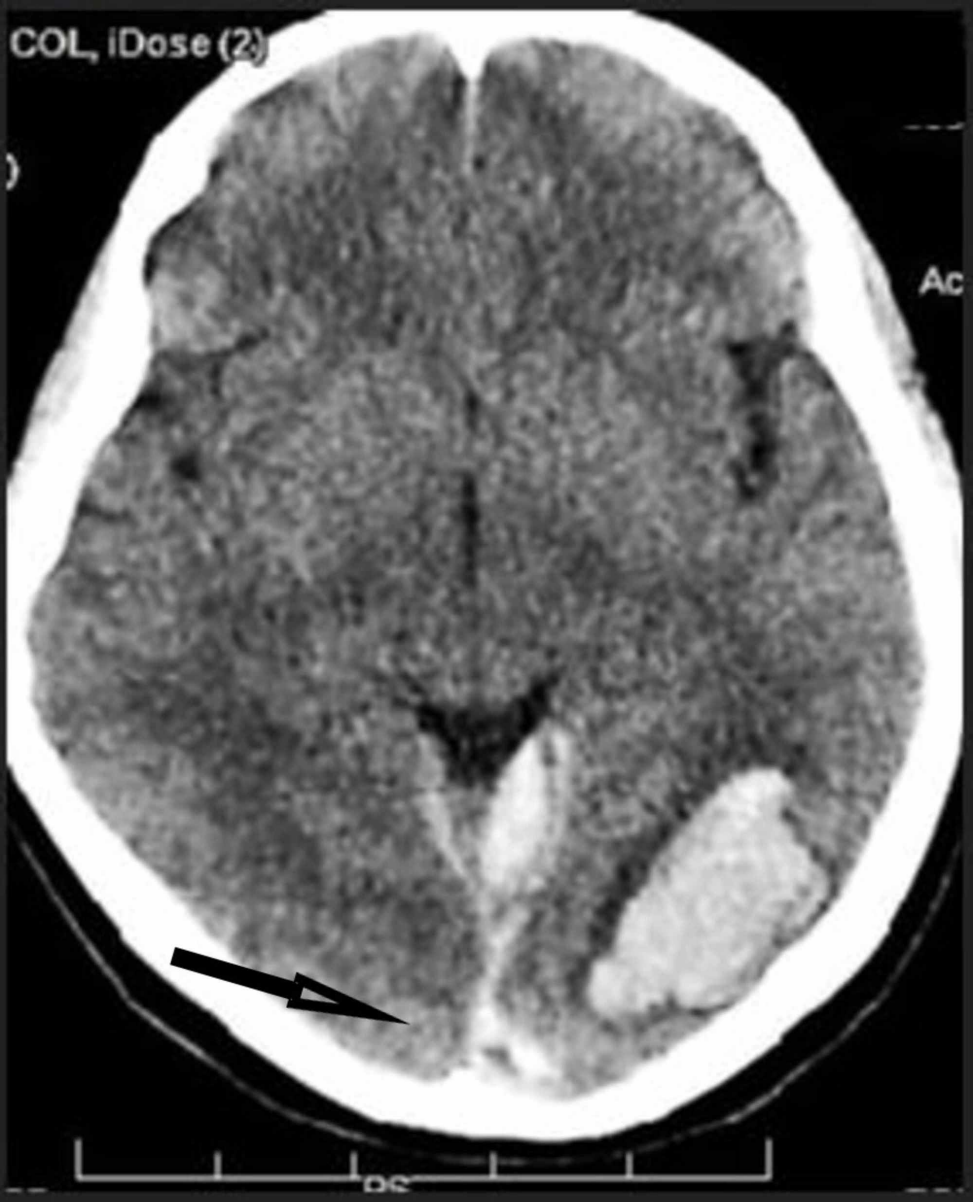
Computed tomography without contrast showing left occipital lobe intracerebral hemorrhage, with subdural extension (arrow)

The following evening on March sixth, the patient suffered the sudden onset of the worst headache of her life with vomiting, then right leg weakness, “hip numbness”, difficulty walking (“her right leg gave out”), and brief loss of consciousness after she was helped to a couch. She was taken to the community hospital emergency department by ambulance, where her temperature was 36.9 °C (98.4 °F), pulse 116 beats/minute, blood pressure 125/72 mmHg, respirations 20 breaths/minute, and saturation 95% on room air, with no heart murmur heard, a few pulmonary rhonchi reported, and neurological examination reported as normal, with no abnormalities of mental status, pupillary reaction to light, cranial nerves, sensation or motor strength. Her hemoglobin was 9.5 g/dL, white blood cell count 14,200/cu mm (82% neutrophils, 11% lymphocytes, 6% monocytes, 1% eosinophils), platelets 389,000/cu mm, international normalized ratio 1.13, partial thromboplastin time 25 seconds, glucose 106 mg/dL, troponin <0.01 ng/mL and pro-brain natriuretic peptide 653 pcg/mL. She had sinus tachycardia on electrocardiogram. Portable chest radiograph showed diffuse ground-glass opacity in the right lung (Figure 3). Computed tomography of the head without contrast showed a 4.5 x 2.2 x 2.3 cm parenchymal hemorrhage in the left occipital lobe, estimated volume 11.4 mL, with subdural extension up to 0.8 cm thick on the left and up to 0.6 cm thick on the right, extending into the upper cervical spinal canal (Figure 4). The patient was started on levetiracetam, piperacillin-tazobactam, and vancomycin; she was then transferred to a larger hospital.

On admission to the larger hospital, no heart murmur was heard, the patient’s chest was reportedly clear, and she was alert and oriented. Her face was symmetric, her speech clear, her pupils 3 mm and reactive, handgrip strong bilaterally, upper extremity strength normal bilaterally, lower extremity strength “moderate” bilaterally, upper extremity sensation normal bilaterally, lower extremity sensation “full” bilaterally, and cranial nerves normal; gag, cough and bilateral corneal reflexes were present and Babinski signs absent. On hospital day two, magnetic resonance imaging and computed tomography angiography confirmed the findings on the non-contrast computed tomography.

On hospital day three, cerebral angiogram showed a small 0.2 cm aneurysm of an abnormal distal left posterior cerebral artery vessel; the patient was taken to surgery, but the aneurysm was already occluded and there was no flow into the abnormal vessel. Blood cultures from the community hospital on day one were positive for Gram-positive cocci. Piperacillin-tazobactam and vancomycin were continued. Transthoracic echocardiogram showed severe mitral stenosis and regurgitation.

**Figure 5:**
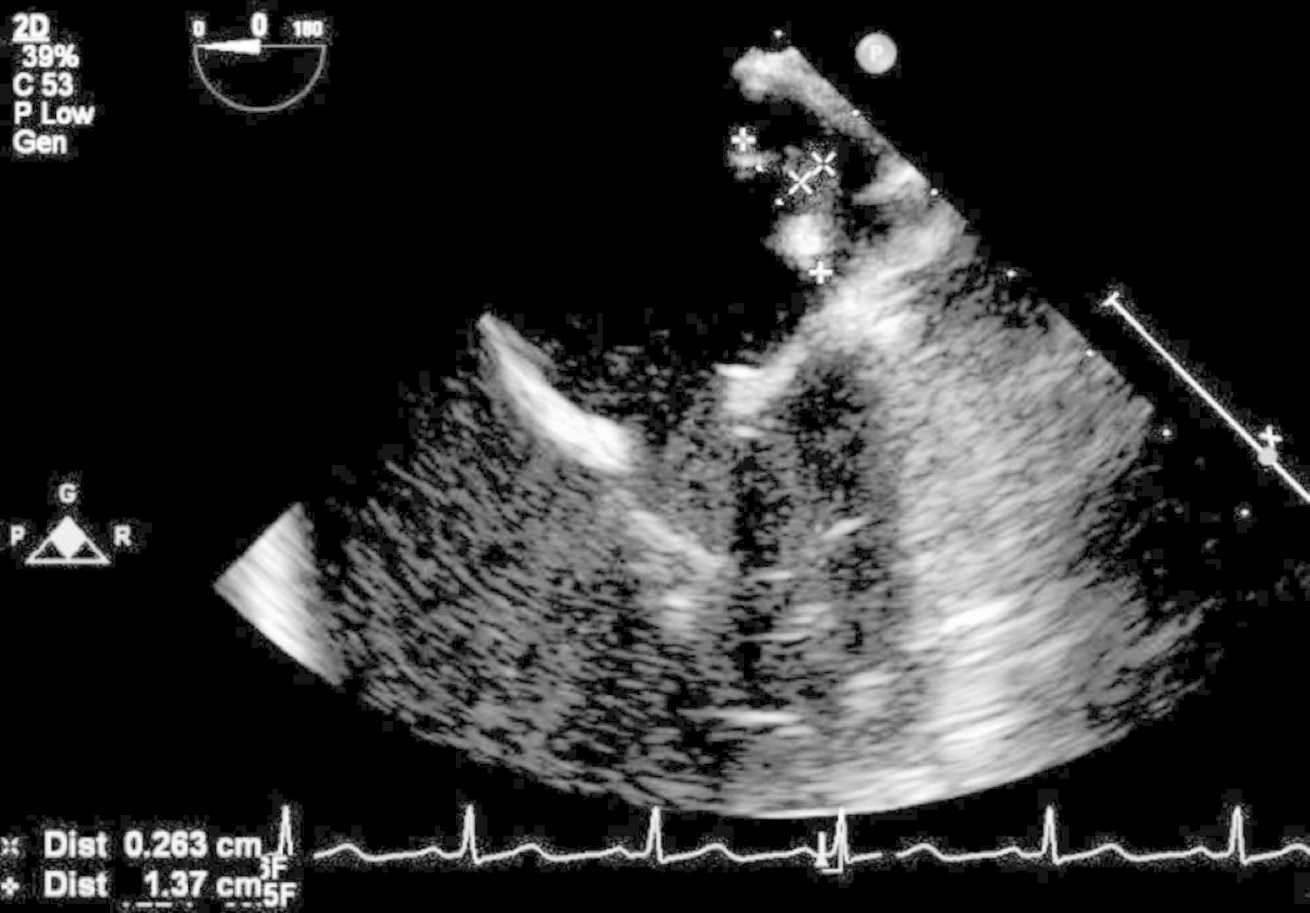
Transesophageal echocardiogram showing 1.37 x 0.263 cm pedunculated mass on the posterior wall of the left atrium, indicated by the caliper marks for measuring it, in the upper portion of the image

**Figure 6:**
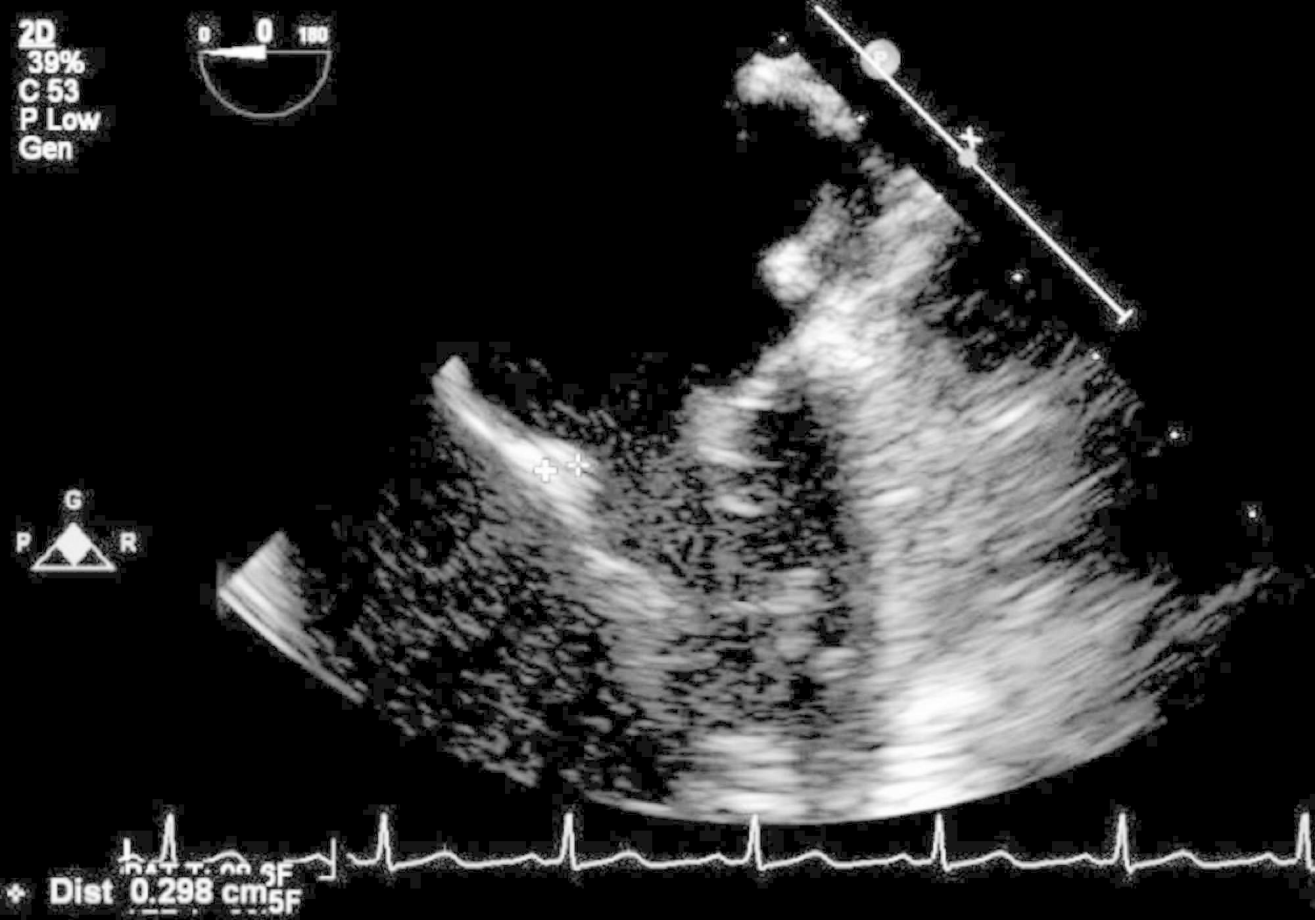
Transesophageal echocardiogram showing 0.298 cm lobule on the anterior leaflet of the mitral valve, indicated by the caliper marks for measuring it, in the lower left portion of the image

On hospital day four, cardiology was consulted and a cardiologist heard a grade 3/6 holosystolic murmur, best heard at the apex. A history of a heart murmur as a child was elicited from the family. Transesophageal echocardiogram showed a 1.37 x 0.263 cm pedunculated mass on the posterior wall of the left atrium (Figure 5), a 0.298 cm lobule on the anterior leaflet of the mitral valve (Figure 6), a stalk on the posterior leaflet of the mitral valve and severe mitral regurgitation. Occupational therapy was consulted and a right superior temporal visual field cut was diagnosed.

On hospital day six, the blood culture isolate was identified as a viridans streptococcus. Ceftriaxone was substituted for piperacillin-tazobactam and vancomycin. History of Janeway lesions was added to the patient’s problem list. On hospital day eight, a dentist consultant elicited a history of severe dental infection requiring the extraction of multiple teeth. Follow-up cerebral angiogram was all negative. On day 10, the patient was discharged with a principal diagnosis of endocarditis, a diagnosis of non-rheumatic mitral valve insufficiency, and ongoing intravenous ceftriaxone therapy.

Ten weeks later, on May 24, the patient underwent minimally invasive mitral valve repair with P2 resection, P1-P3 sliding plasty and placement of a saddle ring, which was successful. One year later, the patient was alive and well, in regular follow-up care.

## Discussion

This patient presented with classic features of subacute bacterial endocarditis due to viridans streptococci invading the bloodstream from an oral source of poor dentition and infecting a presumably abnormal mitral valve, but the diagnosis eluded multiple clinicians over a course of seven months. How did this happen and what can we learn to improve patient safety from the analysis of this case? The six most salient clinician encounters in which the providers failed to make the diagnosis of infective endocarditis are detailed above. Analgesic medication, inadequate physical examination and insufficient differential diagnosis each apparently contributed to the delayed diagnosis.

Fever is present as a manifestation of infective endocarditis in approximately 90% of cases [3]. Fever was absent in this case every time the patient presented most likely because she was taking ibuprofen and acetaminophen. It is possible, however, that the patient was having a fever at times of day other than the times she presented because fever is often intermittent. A thorough review of systems, carefully questioning and listening to a patient, can potentially reveal a subtle history of intermittent fever.

A heart murmur is present as a manifestation of infective endocarditis in approximately 85% of cases [3]. This patient had a heart murmur as a child before she had endocarditis as a young adult and native valve endocarditis with low virulence organisms such as viridans streptococci occurs most often on structurally abnormal valves. So it is likely this patient had a heart murmur that was missed by inadequate physical examination. Notably, two weeks before her stroke from the rupture of a small cerebral artery (likely from a small mycotic aneurysm), a nurse asked the patient if she had a heart murmur while auscultating her heart. This suggests that the nurse heard the murmur and then dismissed hearing it. The nurse may have dismissed hearing the murmur from lack of confidence in her auscultation or from this finding not fitting in her narrowly focused differential diagnosis. The reason the clinicians in other encounters missed the murmur and the diagnosis could be the narrow focus of their thinking.

Narrow focus thinking was evident when the patient was hospitalized two days before her stroke with sudden onset epigastric pain, associated with nausea, vomiting, and weight loss. The focus of evaluation in this hospitalization was centered on gastroenterology without taking into account the whole clinical picture. While gastritis from over-the-counter non-steroidal anti-inflammatory medications may have been a good working diagnosis for the patient’s gastrointestinal complaints, the physicians passed over the question of why such a young person took high doses of ibuprofen for months. Similarly, when the patient was seen in clinic for dizziness and diagnosed with vertigo, two weeks before her stroke, the provider did not address this in the setting of an illness that had persisted for months. Similarly, when the patient was seen in the emergency department with sudden onset left flank pain, three weeks before her stroke, the narrow focus was on her left kidney. Additionally, when the patient was seen in the emergency department, six weeks before her stroke, with intermittent extremity pain, swelling and numbness, the narrow focus was on her extremities. When the patient was seen in clinic eight weeks prior to her intracranial hemorrhage with a five-month history of unexplained painful episodes with no end in sight, she was understandably anxious and the narrow focus was on her anxiety and not the cause of it. When analyzing all of the encounters, in this case, the common thread was a narrow focus on the chief complaint without considering it in the larger picture of the patient as a whole person.

It is noteworthy that the patient’s grade 3/6 holosystolic heart murmur was not noted until an echocardiogram had detected severe mitral regurgitation. Similarly, the history of dental disease was not elicited until the organism causing the infective endocarditis was identified as a viridans streptococcus. Medical students are taught that laboratory investigations should be done to confirm diagnoses made by history and physical examination, but this case shows the opposite, which correlates with a long delay in diagnosis.

Infective endocarditis is a dangerous condition where early recognition and treatment are crucial. With accurate diagnosis and treatment in a timely manner, approximately two-thirds of patients will survive [1]. Early diagnosis is the key to improving outcomes in infective endocarditis. As with most disease processes, a thorough history and careful physical examination are both crucial in quickly making an accurate diagnosis. Classically, patients present with fever and heart murmur in the setting of risk factors such as recent dental procedure, poor dental hygiene or intravenous drug use. Over-the-counter anti-inflammatory medications can mask fever. Inadequate physical examination may not detect heart murmurs. Osler nodes, Janeway lesions, and Roth spots are more specific, but infrequent findings [3]. If clinicians are not judicious with their physical examination, they may misinterpret a classic finding. Physicians must always keep a high index of suspicion for infective endocarditis in the appropriate clinical scenario.

## Conclusions

This was a classic case of subacute bacterial endocarditis with Osler nodes and Janeway lesions due to viridans streptococci from an oral source of poor dentition, infecting a presumably abnormal mitral valve. The diagnosis was missed repeatedly by multiple different clinicians over the course of seven months, partly due to missing important details of history and findings of physical examination, attributable to narrow, problem-focused thinking. In addition, the patient took large doses of over-the-counter analgesic medication, masking the most common symptom and sign of infective endocarditis. The images of this case can serve as a reminder of the features of infective endocarditis: an important diagnosis that shouldn't be missed. It is fatal if untreated, but most often successfully treatable, if recognized. Physicians must maintain a high index of suspicion for infective endocarditis.
